# Tracking Progress Towards the Sustainable Development Goals in Four Rural Villages in Limpopo, South Africa

**DOI:** 10.5334/aogh.3139

**Published:** 2021-02-15

**Authors:** Bianca Wernecke, Angela Mathee, Zamantimande Kunene, Yusentha Balakrishna, Thandi Kapwata, Mirriam Mogotsi, Neville Sweijd, Noboru Minakawa, Caradee Yael Wright

**Affiliations:** 1 Environment and Health Research Unit, South African Medical Research Council, Johannesburg, South Africa; 2 Environmental Health Department, Faculty of Health Sciences, University of Johannesburg, Johannesburg, 2028, South Africa; 3 Environmental Health Department, Faculty of Health Sciences, University of Johannesburg, South Africa; 4 School of Public Health, Faculty of Health Sciences, University of the Witwatersrand, Johannesburg, South Africa; 5 Biostatistics Unit, South African Medical Research Council, Durban, South Africa; 6 Applied Centre for Climate and Earth Systems Science, National Research Foundation, Cape Town, South Africa; 7 Institute of Tropical Medicine, Nagasaki University, Nagasaki, Japan; 8 Environment and Health Research Unit, South African Medical Research Council, Pretoria, South Africa; 9 Department of Geography, Geoinformatics and Meteorology, University of Pretoria, Pretoria, South Africa

## Abstract

**Background:**

Measuring national progress towards the Sustainable Development Goals (SDGs) enables the identification of gaps which need to be filled to end poverty, protect the planet and improve lives. Progress is typically calculated using indicators stemming from published methodologies. South Africa tracks progress towards the SDGs at a national scale, but aggregated data may mask progress, or lack thereof, at local levels.

**Objective:**

To assess the progress towards achievement of the SDGs in four low-income, rural villages (Giyani) in South Africa and to relate the findings to national SDG indicators.

**Methods:**

Using data from a cross-sectional environmental health study, the global indicator framework for the SDGs was applied to calculate indicators for Giyani. Local progress towards SDG achievement was compared with national progress, to contextualize and supplement national scale tracking.

**Findings:**

Village scores were mostly in line with country scores for those indices which were computable, given the available data. Low data availability prevented a complete local progress assessment. Higher levels of poverty prevail in the study villages compared to South Africa as a whole (17.7% compared to 7.4%), high unemployment (49.0% compared to 27.3%) and lack of access to information via the Internet (only 4.2% compared to 61.8%) were indicators in the villages identified as falling far short of the South African averages.

**Conclusions:**

Understanding progress towards the SDGs at a local scale is important when trying to unpack national progress. It shines a light upon issues that are not picked up by national composite assessments yet require most urgent attention. Gaps in data required to measure progress towards targets represents a serious stumbling block, preventing the creation of a true reflection of local and national scale progress.

## Introduction

The United Nations Sustainable Development Goals (SDGs) are international guidelines which aim to move the world towards a sustainable future, balancing economic growth, social development, and environmental protection [1,2]. They build upon the Millennium Development Goals (MDGs) and are set to be achieved by 2030. Though each of the 17 goals has well-defined targets (Goal 3, for instance, speaks to the promotion and protection of human health and well-being), all goals are interlinked, and progress towards the one is best achieved in conjunction with progress towards others [3]. The achievement of the SDGs is particularly relevant to low- and middle-income countries (LMICs) such as South Africa, where large strides towards meeting these goals still need to be made.

For regions, countries, districts, cities, and towns to meet the SDGs, specific and locally relevant strategies, policies and programs need to be designed and implemented [4]. The African Union has developed the “Agenda 2063” which is a strategic framework for socio-economic transformation of the African continent over the next 50 years [4]. It seeks to accelerate the implementation of past and existing continental initiatives for growth and sustainable development [5]. Previously, Agenda 2063 tracked its progress against the MDGs. Going forward, it tracks its progress against the SDGs [4]. An example of a more country-specific framework is Kenya’s “Vision 2030” which aims to transform the country into a newly industrializing, middle-income country providing a high quality of life to all its citizens in a clean, secure environment by 2030 [6].

In South Africa, the National Development Plan (NDP) was adopted in 2012 and defines national development priorities for the country. Despite the NDP pre-dating the SDG process by several years, there is nevertheless around 74% convergence between the two frameworks (***Table 1***) illustrating the relevance of the SDG process for South Africa [13].

**Table 1:** Synergies between the SDGs and South Africa’s NDP (adapted from Cumming et al., 2017 and The SDG Country Report 2019 – South Africa) [7,13].


SUSTAINABLE DEVELOPMENT GOAL (SDG)		SOUTH AFRICAN NATIONAL DEVELOPMENT PLAN (NDP) FOCUS AREAS

1.	End poverty in all its forms everywhere.	Chapter 3: Economy and employment Chapter 4: Economic infrastructure Chapter 5: Environmental sustainability and resilience Chapter 6: Inclusive rural economy Chapter 8: Transforming human settlements Chapter 11: Social protection

2.	End hunger, achieve food security and improved nutrition, and promote sustainable agriculture.	Chapter 3: Economy and employment Chapter 4: Economic infrastructure Chapter 5: Environmental sustainability and resilience Chapter 6: Inclusive rural economy Chapter 7: Positioning South Africa in the world Chapter 10: Healthcare for all Chapter 11: Social protection

3.	Ensure healthy lives and promote well-being for all ages.	Chapter 5: Environmental sustainability and resilience Chapter 10: Healthcare for all Chapter 12: Building safer communities

4.	Ensure inclusive and equitable quality education and promote life-long learning opportunities for all.	Chapter 9: Improving education, training, and innovation Chapter 11: Social protection

5.	Achieve gender equality and empower all women and girls.	Chapter 4: Economic infrastructure Chapter 6: Inclusive rural economy Chapter 10: Healthcare for all Chapter 12: Building safer communities

6.	Ensure availability and sustainable management of water and sanitation for all.	Chapter 4: Economic infrastructure Chapter 5: Environmental sustainability and resilience Chapter 8: Transforming human settlements

7.	Ensure access to affordable, reliable, sustainable, and modern energy for all.	Chapter 4: Economic infrastructure Chapter 5: Environmental sustainability and resilience

8.	Promote sustained, inclusive, and sustainable economic growth, full and productive employment, and decent work for all.	Chapter 3: Economy and employment Chapter 5: Environmental sustainability and resilience Chapter 6: Inclusive rural economy Chapter 9: Improving education, training, and innovation

9.	Build resilient infrastructure, promote inclusive and sustainable industrialization and foster innovation.	Chapter 4: Economic infrastructure Chapter 9: Improving education, training, and innovation

10.	Reduce inequality within and among countries.	Central theme of NDP Chapter 3: Economy and employment Chapter 11: Social protection Chapter 12: Building safer communities Chapter 15: Nation building and social cohesion

11.	Make cities and human settlements inclusive, safe, resilient, and sustainable.	Chapter 4: Economic infrastructure Chapter 5: Environmental sustainability and resilience Chapter 8 Transforming human settlements Chapter 13: Building a capable and developmental state

12.	Ensure sustainable consumption and production patterns.	Chapter 4: Economic infrastructure Chapter 5: Environmental sustainability and resilience Chapter 6: Inclusive rural economy Chapter 8: Transforming human settlements

13.	Take urgent action to combat climate change and its impacts.	Chapter 4: Economic infrastructure Chapter 5: Environmental sustainability and resilience

14.	Conserve and sustainably use the oceans, seas, and marine resources for sustainable development.	Chapter 5: Environmental Sustainability and resilience

15.	Protect, restore, and promote sustainable use of terrestrial ecosystems, sustainably manage forests, combat desertification, and halt and reverse land degradation and halt biodiversity loss.	Chapter 5: Environmental sustainability and resilience

16.	Promote peaceful and inclusive societies for sustainable development, provide access to justice for all and build effective, accountable, and inclusive institutions at all levels.	Chapter 11: Social protection Chapter 12: Building safer communities Chapter 13: Building a capable and developmental state Chapter 14: Fighting corruption Chapter 15: Notion building and social cohesion

17.	Strengthen the means of implementation and revitalize the global partnership for sustainable development.	Chapter 7: Positioning South Africa in the world


*Note*: This list is not exhaustive.

### Tracking progress towards meeting the SDGs

In 2019, *The Bertelsmann Stiftung* and *The Sustainable Development Solutions Network* co-produced a 2019 SDG Index and Dashboards report [8]. This report gives annual overviews of countries’ performance against the 17 SDGs, basing its findings on publicly available data published by key international institutions (World Bank, WHO, ILO etc.) and organizations, including research centers and non-governmental organizations. In 2019, Denmark, Sweden and Finland topped the index charts with good performance towards achieving the SDGs, whereas the Democratic Republic of Congo, Chad and the Central African Republic ranked last among the 162 countries assessed. South Africa placed 113^th^ on the list.

A similar exercise was conducted for Africa specifically, by *The Sustainable Development Goals Centre for Africa* and *Sustainable Development Solutions Network*, where South Africa ranks 10^th^ out of 52 African countries assessed (top ranked in Africa in 2019 was Mauritius, a country deemed two thirds of the way towards achieving the SDGs) [9].

South Africa’s dashboard in 2019 reported the country’s average performance (yellow, orange, or red) on each of the SDGs (***Table 2***). In 2019, South Africa did not fully achieve any of the SDGs. This may be attributed to a lack of actual progress towards goal achievement or to inadequate sources of information or data to assess real progress.

**Table 2:**
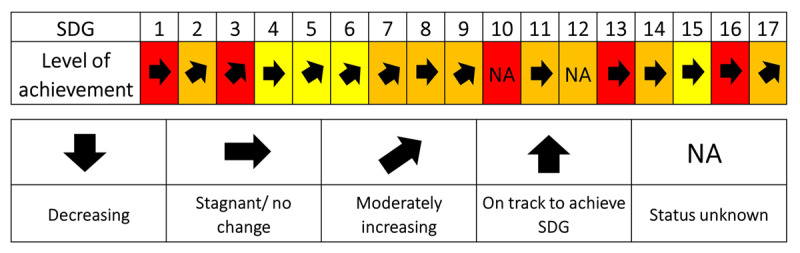
South Africa’s SDG dashboard for 2019 showing a performance assessment towards the 17 SDGs and associated trends. * Adapted from *The Sustainable Development Goals Centre for Africa and Sustainable Development Solutions Network 2019*. *Note*: There are four color categories. Green denotes SDG achievement, followed by yellow and orange which indicate an increasing distance from SDG achievement. Red highlights major challenges.

In 2017, South Africa was able to report on 66% of the SDGs’ social targets (SDGs 1–5 and 11), 73% of the economic targets (SDGs 7–10 and 12), 57% of the environmental targets (SDGs 6 and 13–15), 73% of the peace and security targets (SDG 16) and 29% of the targets grouped under the 17th SDG which is associated with the means of overall implementation towards achieving the goals [10].

These statistics are useful to understand how well certain locations in South Africa are faring towards SDG achievement especially when compared to data nationally and from other countries. The country’s first full-scale SDG progress tracking report was compiled by Statistics South Africa in 2017 and outlined a National SDG Indicator Framework. The report illustrated progress towards selected 2030 targets as specified by the SDGs and their published indices [3,11]. Most of the 232 SDG indicators have internationally established and agreed upon standards and methodologies for computation (Tier I and II SDG indicators), with only a few indicators for which the methodology or standards are still being developed or tested (Tier III SDG indicators) [12]. In its 2019 Country Report, South Africa was able to report on 128 of the 232 indicators. Tracking progress towards SDG goals, targets and indicators is complex as the technical process of developing methodologies to measure some of the proposed indicators, especially the non-quantitative indices, is a constant work in progress [11,13].

### The benefit of tracking goals locally

Success towards meeting goals and targets is typically measured at a national scale to enable a comparison towards global progress. However, tracking smaller scale, local progress assists in better identifying and understanding gaps that need to be addressed to meet the targets within a country’s own borders. When considered at a global scale, African countries frequently appear as “red” (meaning major challenges exist) in the global SDG tracking dashboards, even though work is being done in those countries to progress. A higher resolution assessment at local scale, instead of a national scale, could help highlight progress made towards SDG target achievement which would ordinarily be masked by an amalgamated national index. Showcasing local government efforts could help illustrate whether countries are on the appropriate track towards meeting the SDGs and provide a basis for shared experiences and best practices [9].

The aim of this study was to assess progress towards achievement of the SDGs in four low-income, rural villages in South Africa. Findings are compared with national indicators where possible. Results of this exercise help to identify data gaps, areas for potential research and development, as well as to stimulate discussion about the types of data needed for SDG target and index computation for South Africa. This work provides a model for how local-scale assessments can be approached and how the results can then be contextualized within national progress.

## Methods

### Study site and data collection

**Figure 1:**
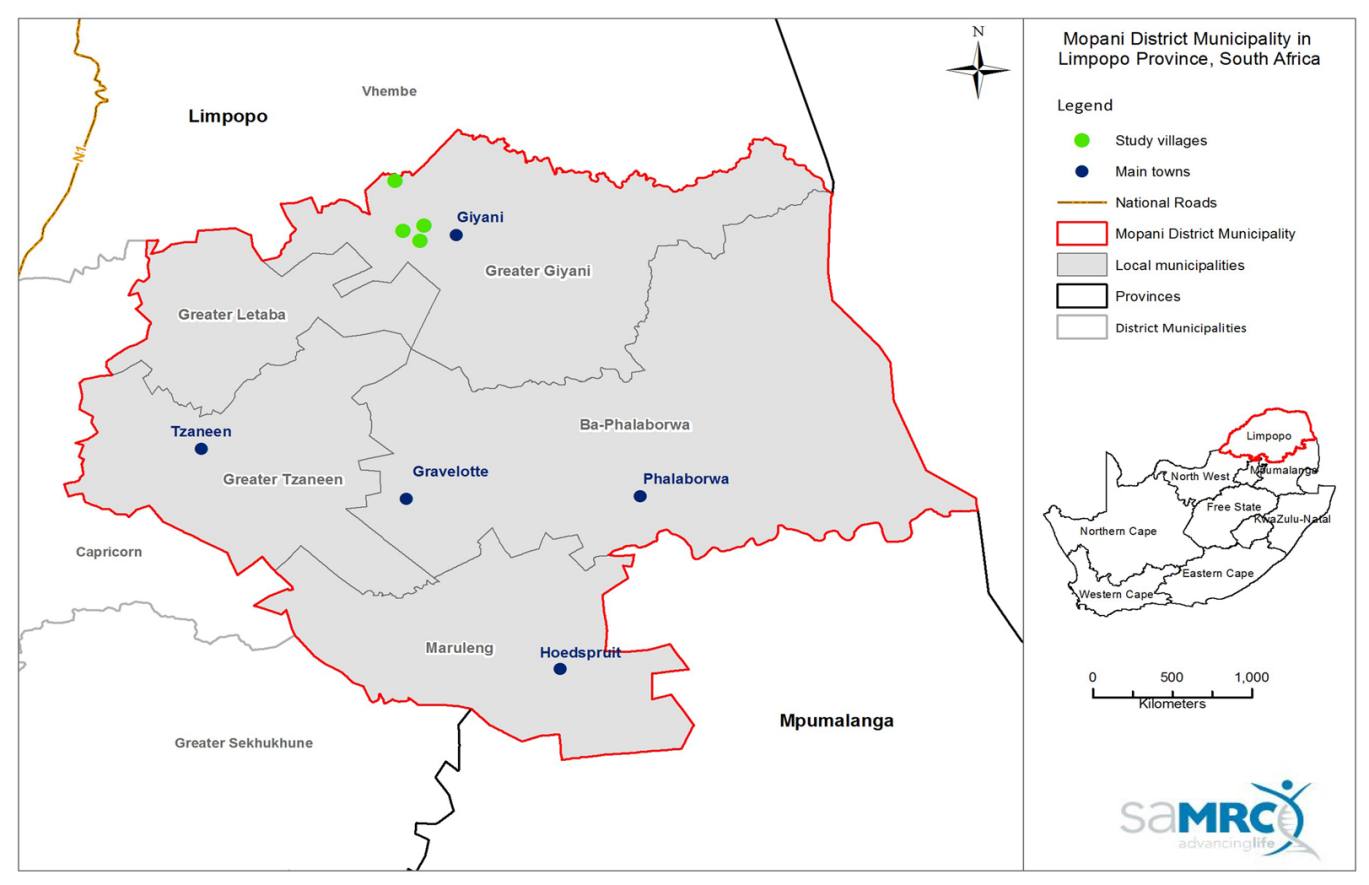
Location of the villages included in the study area surrounding the town of Giyani, Limpopo.

A cross-sectional survey was used to collect data from 400 dwellings in the greater Giyani local municipality located in Limpopo Province, South Africa in September 2016 (***Figure 1***). The municipality has a population size of 247,657 inhabitants, living in 57,537 households, the majority of which are clustered in hamlets or villages. Dwellings for the study were randomly selected from four villages. A cluster sampling method was used to select 100 households in each village. Ethics clearance for the study was granted by the South African Medical Research Council Research Ethics Committee (Certificate clearance no. EC005-3/2014, 9 May 2017) and permission was sought from the provincial, local and traditional leadership of the study areas.

### Questionnaire and data analysis

Following written informed consent, a structured questionnaire was administered by a trained field worker to a respondent from each of the 400 households participating in the study. A household member of at least 18 years of age provided socio-economic and demographic information for their household (defined as the people who eat a meal together). Questionnaire data were double-data-entered into Microsoft Excel and analyzed for descriptive frequencies using STATA version 14.

### Comparison of Giyani results with SDG targets and indices

**Figure 2:**
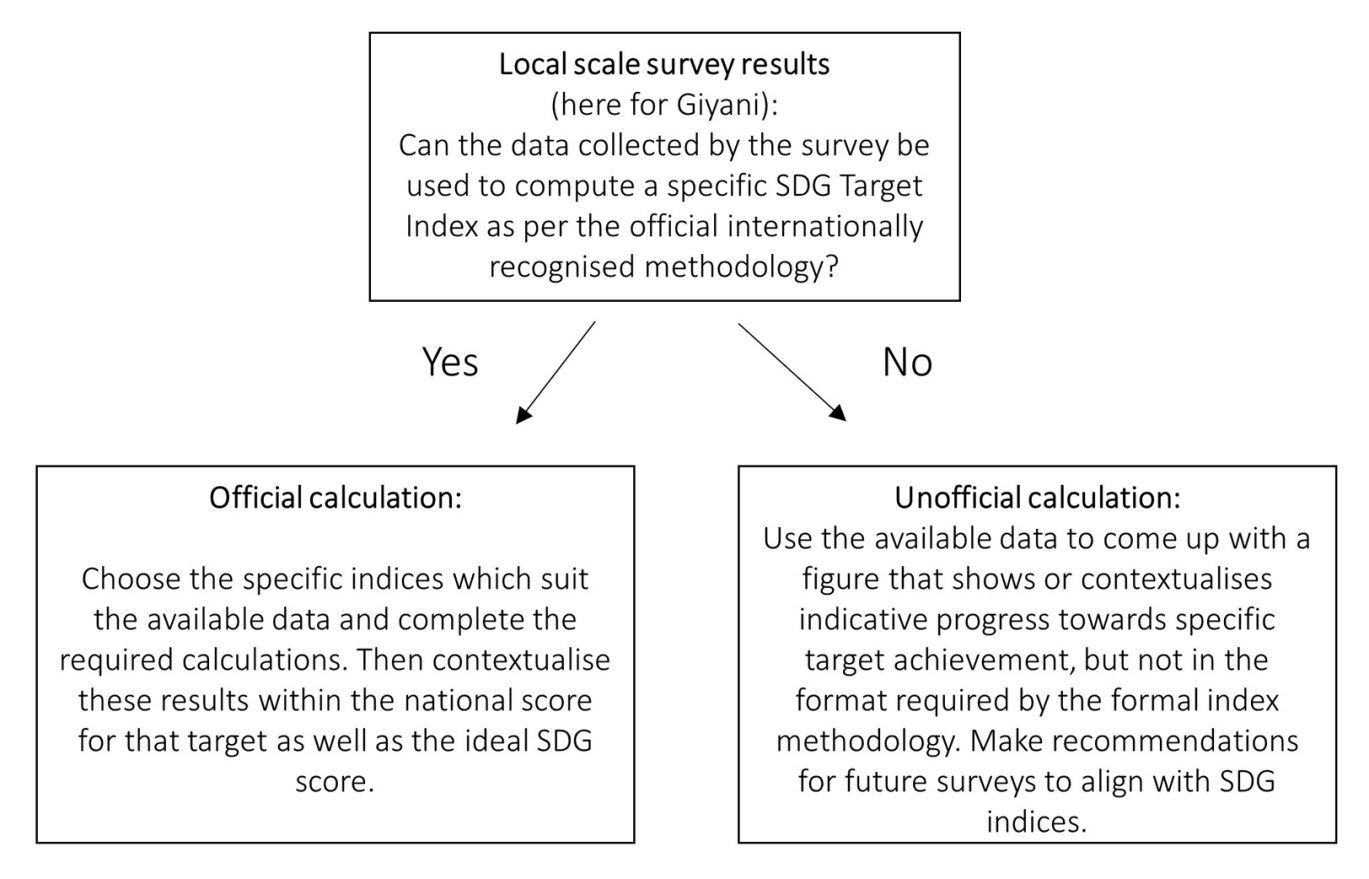
Model used to assess local survey data within the context of the SDGs either officially or unofficially.

Responses to questions in the Giyani study questionnaire which aligned with SDG indicators were used to calculate a Giyani-specific index which was then compared to the corresponding index listed in the South African Country Report [12]. Existing reports focusing on South Africa’s progress towards the SDGs targets and indices were used to contextualize the results from the Giyani study [8,11,12]. Where responses matched SDG indices, a direct comparison was possible. This was mostly the case for *relative* indices, e.g. percentage of population living below the international poverty line. Indicative comparisons were made for *absolute* indices, e.g. total number of social grants received. An overview of the model used is illustrated in ***Figure 2***.

## Results and Discussion

Data collected from four villages in rural Giyani were compared to 20 SDG indicators using the model illustrated above (Indicators falling under SDGs 1, 6, 8, 11 and 17 covering issues of no poverty, clean water and sanitation, decent work and economic growth, sustainable cities and communities as well as partnerships for the goals). Of these 20 indicators, seven were directly calculated according to officially recognized Tier I and Tier II computation methods and 13 were used for contextualization purposes only (***Table 3***).

**Table 3:**
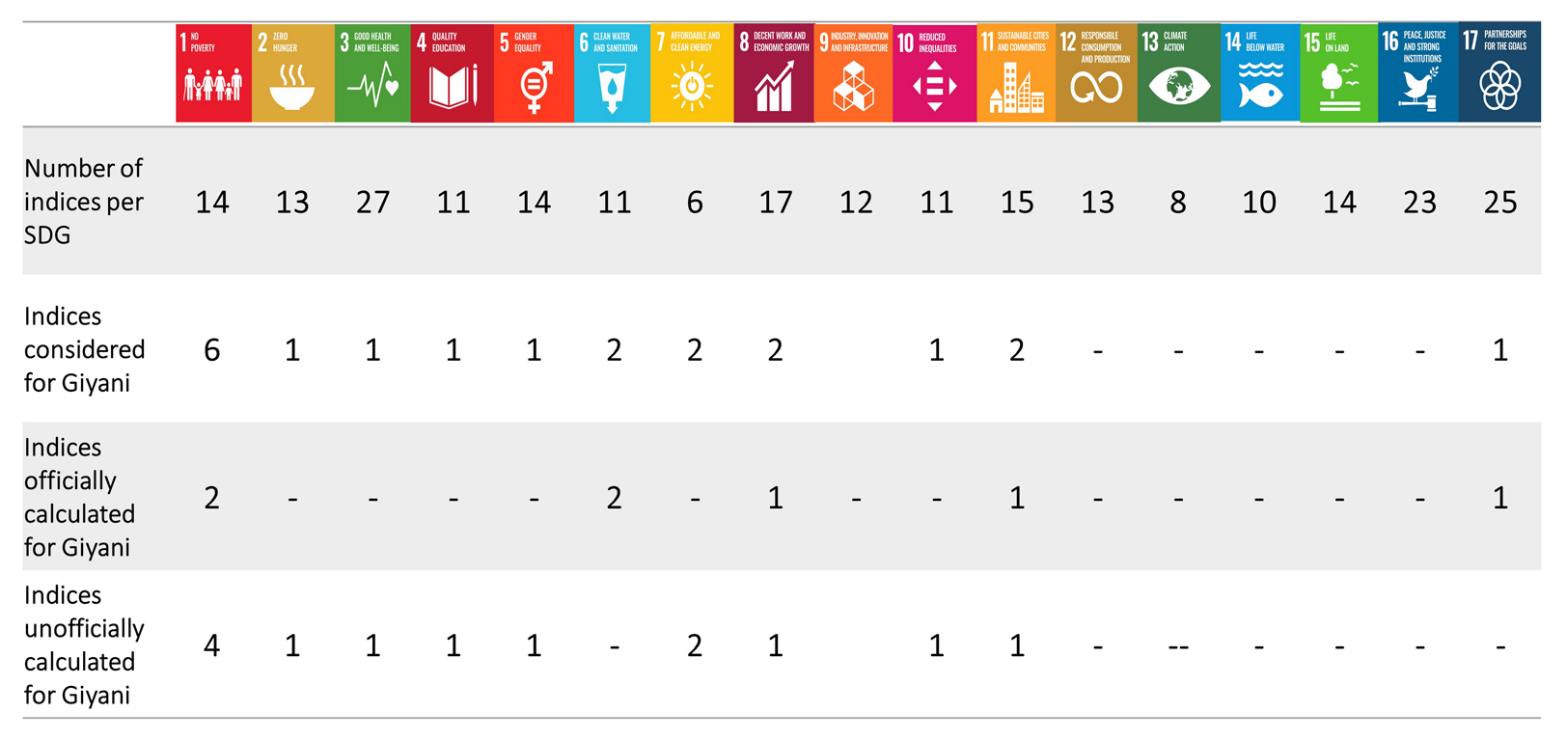
Overview of the number of SDG indices considered, officially and unofficially, for this study using data collected in Giyani villages.

### Direct comparison of Giyani and South African SDG indicator scores (Using South African reports as a baseline)

The income of nearly one third of Giyani’s population ranks below the international and the national poverty lines (***Table 4***). The proportion of the Giyani population (17.7%) with an income below the international poverty line exceeds the national proportion which is 7.4%. Compared to South Africa as a whole (25.2%), a smaller proportion of people lives with an income below the national poverty line (17.7%). The unemployment rate in Giyani (49.0%) was higher than the figure reported for South Africa (27.3%) [13]. Given the definition of access to clean and affordable drinking water and sanitation, as outlined by the SDG South Africa Baseline Report (percentage of population using an improved basic drinking water source, i.e. piped water into dwelling, yard or plot; public taps or standpipes; boreholes; protected dug wells; protected springs and rainwater and the total number of population using improved sanitation: flush or pour flush toilets to sewer systems, septic tanks or pit latrines, ventilated improved pit latrines, pit latrines with a slab, and composting toilets), data suggested that Giyani villages fared well in these water-focused indices as almost 100% of the surveyed households reported to have some form of water access or sanitation [11,13]. Generally, it seemed that more people living in Giyani live in formal dwellings, when compared to the rest of the country. Fewer people (4.2%) were reported to have access to the Internet in Giyani compared to the rest of the country (61.8%).

**Table 4:** Officially calculated SDG indicator scores (%) for South Africa vs Giyani villages in relation to the ideal SDG targets, categorized according to relevant socioeconomic status metric.


SES METRIC	INDICATOR (SDG)	SOUTH AFRICA (%)	GIYANI (%)	TARGET (%)	CONTEXT

Income	Population living under international poverty line (SDG 1–1.1.1).	7.4	17.7	0.0	1) No income = 17.7% 2) <R 1,000.00 = 29.8% At least 17.7% of respondents lived below the 2015 international poverty line at the time of the survey (R 456.38/person/month), as they did not earn a salary at all. The percentage could be higher, given that 29.8% of respondents earned less than R 1,000.00/ month (excluding any possible grants received by the household). Exact figures for respondents’ incomes were not available, as data on income were collected as an ordinal variable.

**Income**	**Population living under national poverty line (SDG 1–1.2.1)**.	**25.2**	**17.7**	**0.0**	**1) No income = 17.7%** **2) <R 1000 = 29.8%** **17.7% of respondents lived below the food poverty line of R441/person/month, as they did not receive any income at all. The proportion of people living below the poverty line could be marginally higher, considering that an additional 29.8% of people earned below R 1,000.00/ month. Exact figures for respondents’ incomes were not available, as data on income were collected as an ordinal variable**.

Employment	Unemployment rate (SDG 8–8.5.2).	27.3	49.0	0.0	The unemployment rate in Giyani was almost double the national rate.

**Water**	**Access to drinking water (SDG6–6.1.1)**.	**86.0**	**99.3**	**100.0**	**99.3% of households had access to a basic drinking water source. This includes piped water into dwelling, yard, or plot; public taps or standpipes; boreholes or tube wells; protected dug wells; protected springs and rainwater. In Giyani, while many households had access to piped water, the water systems were unreliable and households often had extended periods of time without running water, relying on water storage, leading to other health-related risks (e.g., bacteriological contamination causing diarrheal disease) [14]**.

**Sanitation**	**Access to sanitation (SDG 6–6.2.1D)**.	**70.0**	**99.3**	**100.0**	**99.3% of households had access to “improved sanitation” facilities (flush or pour flush toilets connected to sewer systems, septic tanks, or pit latrines, ventilated improved pit latrines, pit latrines with a slab, and composting toilets). This is more than the reported national figure**.

**Housing**	**Population living in informal dwellings (SDG 11–11.1.1)**.	**12.2**	**2.9**	**0.0**	**2.9% of participating households lived in informal dwellings. This means that more people in Giyani live in formal dwellings, compared to the rest of the country**.

Access to internet	Proportion of people using the internet (SDG 17–17.8.1).	61.8	4.2	100.0	4.2% of households had access to internet. This is substantially less than the country’s measurement.


Bold: Better than national score. *Note*: for more information on results and computation, see Table S1 in supplementary tables.

### Contextualization of further Giyani results with the SDGs using unofficial methods

Two supplementary tables illustrate progress towards the SDGs in Giyani villages in relation to the rest of the country. Table S1 lists those indices for which data from the Giyani survey were available for comparison with SDG target indices. Results from the Giyani villages were also compared to South Africa’s outcomes, where the colors red, orange or green were allocated to each score (Table S1). The colors translate to “Giyani is doing worse than the rest of South Africa in this index”, “Giyani is on par with the rest of South Africa in this index” and “Giyani scores better than South Africa in this index”, respectively.

Table S2 highlights instances in which the Giyani survey results did not provide enough information to officially calculate a given index, or where there is no existing South African score available to make a comparison, but where results are nevertheless of contextual value in relation to the SDGs. Table S2 is useful as a broad indication of how Giyani is faring compared to the rest of the country in relation to a particular index. For instance, the assessment illustrated that more than two thirds of households sampled received income through a state grant (69.5% of households received at least one child grant, 43.4% received at least one old age grant, 4.2% received a disability grant and 2.7% benefited from other grants).

Giyani is grappling with many of the same socio-economic issues which persist in other parts of South Africa. Poverty affects almost a third of households. Access to drinking water and sanitation, though “present”, is not always reliable or safe, and requires urgent attention [14]. Unemployment is high with almost half of households listing at least one household member as unemployed, and internet access, an essential amenity for meaningful participation in today’s economy, is not nearly at a desired level.

### Study limitations

Here we underline the challenges associated with holistic, local and national assessments of SDG progress, including masking of extreme under-development in a society as unequal as South Africa. Data collected in Giyani yielded insights on various socio-economic issues covered by the SDGs. The original study survey was not designed for the primary purpose of an SDG progress assessment, and so the data were mostly not useful according to the globally accepted official calculation methodologies. This is frequently a pitfall in project-specific surveys: they are often not created with other assessments in mind. As data gaps represent one of the main reasons why progress towards the SDGs in RSA cannot be adequately computed and expressed, it is advisable that researchers be mindful of the opportunity for data compatibility between the SDG framework and survey data, for example in respect of socio-economic and human health. If this is done, survey data can contribute to the understanding of local, as well as national, progress towards SDG achievement. This is particularly important in countries where the lack resources focused on socio-economic surveys may also represent a limiting factor into why large data gaps exist.

## Conclusions

This exercise aimed to consider data that were collected in four rural villages in Giyani in relation to the SDG indicators. The results were then contextualized by comparing them to South Africa’s national indicator results. It was found that Giyani villages fared worse than the rest of the country on average, in relation to levels of internationally defined poverty, unemployment, and access to the internet. Giyani villages however, are faring better than the national status quo in terms of nationally defined poverty levels, access to water (albeit that supply and quality may not be consistent) and sanitation, as well as in terms of the number of people living in informal dwellings.

It should be brought to the attention of researchers and survey teams that surveys could be formulated in such a way that the survey results can be directly fed into the calculations of relevant SDG target indices. This would contribute to the improvement of overall data availability for the accurate assessment of how South Africa is progressing towards meeting the SDGs, at both macro- and micro-levels. It would also highlight very specific gaps which need to be targeted at a local level to ensure the SDGs are achieved. South Africa, and indeed small communities like Giyani, are still far from reaching the SDGs, however, tracking their progress using reliable data, provides useful information to guide tailored interventions and awareness campaigns.

## Additional Files

The additional files for this article can be found as follows:

10.5334/aogh.3139.s1 Table S1. Detrended Oscillation and Clock Parameters.

10.5334/aogh.3139.s2 Table S2. Comparing Giyani and South Africa SDG scores for targets for which indices could not be computed for official/direct comparison but were unofficially calculated for contextualization.
