# Supplementary material for: Tracking Progress Towards the Sustainable Development Goals in Four Rural Villages in Limpopo, South Africa
Source: Ann Glob Health. 2021 Feb 15;87(1):16. doi: 10.5334/aogh.3139 (PMC7894382; doi:10.5334/aogh.3139)
Supplement: Table S1. — Detrended Oscillation and Clock Parameters. [file agh-87-1-3139-s1.pdf]

Table S1. Comparing Giyani and South Africa SDG official scores for targets for which indices could be computed *(important definitions presented below table)*

Worse than **On par with** **Better than**

**SDG Target**	**SDG Indicator**	**Method of computation (RSA SDG Baseline report)**	**South African score (based on SDG baseline report)**	**Giyani Questionnaire item(s) providing relevant data in 2017**	**Giyani score**	
**Goal 1. End poverty in all its forms everywhere**						
1.1 By 2030, eradicate extreme poverty for all people everywhere, currently measured as people living on less than $1.25 a day	1.1.1 Proportion of population below the international poverty line, by sex, age, employment status and geographical location (urban/rural)	The number of persons living in households below the international poverty line divided by the total number of persons multiplied by 100	Percentage of population below the international poverty line in 2015:<br><br>7% (Stats SA 2017) using $ 1.25/ day as international poverty line<br><br>19% (Stats SA 2019) using $ 1.9/ day as international poverty line	What is the average monthly income for this household- excluding grant and pensions?	Reponses relevant to indicator:<br>1) No income = 18%<br>2) <R 1000 = 30%<br><br>What does this mean: At least 17.7% of respondents live below the 2015 international poverty line at the time of the survey (R 456.38/ month), as they do not earn a salary at all (excluding grants). The percentage could be higher, given the fact that 29.8% of respondents earn less than R 1000/ month, however, it is not specified how much these respondents earn as the question was multiple-choice in nature, and so	

					exact income numbers were not derived. ($ 1.25 used as international poverty line.	
1.2 By 2030, reduce at least by half the proportion of men, women and children of all ages living in poverty in all its dimensions according to national definitions	1.2.1 Proportion of population living below the national poverty line, by sex and age	The number of persons living in households below the national poverty line divided by the total population	Proportion of population living below the national poverty line in 2015:<br>- UBPL: 0.56<br>- LBPL: 0.40<br>- FPL: 0.25	What is the average monthly income for this household- excluding grant and pensions?	Reponses relevant to indicator:<br>1) No income = 18%<br>2) <R 1000 = 30%<br><br>What does this mean: 17.7% of respondents live below the Food Poverty Line), as they do not receive any income at all (excluding grants). The proportion of people living below the poverty line could be marginally higher, considering that an additional 30% of people earn below R 1000.00/ month. Exact figures for respondents’ incomes were not available, as data on income were collected as an ordinal scale.	

Goal 6. Ensure availability and sustainable management of water and sanitation for all						
6.1 By 2030, achieve universal and equitable access to safe and affordable drinking water for all	6.1.1 Proportion of population using safely managed drinking water services	Percentage of population using an improved basic drinking water source (piped water into dwelling, yard or plot; public taps or standpipes; boreholes or tube wells; protected dug wells; protected springs and rainwater) divided by the total population multiplied by 100.	In 2017 for RSA 86%<br><br>Safely managed: 80%<br>Basic Service: 6%<br>Limited Service: 9%<br>Unimproved: 3%<br>No service: 3%	Where do you mainly get your drinking water?<br>If your main source of drinking water is not available, where do you get your drinking water?<br>Do you store water for drinking or cooking in a container?	99% of households have access to a basic drinking water source.	
6.2 By 2030, achieve access to adequate and equitable sanitation and hygiene for all and end open defecation, paying special attention to the needs of women and girls and those in	6.2.1D: Percentage of population using safely managed sanitation services, including a hand-washing facility with soap and water	The total number of the population using improved sanitation (flush or pour flush toilets to sewer systems, septic tanks or pit latrines, ventilated improved pit	In RSA for 2017:<br><br>Basic service: 70%<br>Limited Service: 13.0%<br>Unimproved: 15%<br>Open defecation: 2%	What type of toilet does your household mainly use?	99% of households have access to "improved sanitation" facilities (flush or pour flush toilets to sewer systems, septic tanks or pit latrines, ventilated improved pit latrines, pit latrines with a slab, and composting toilets).	

vulnerable situations		latrines, pit latrines with a slab, and composting toilets) divided by the total population multiplied by 100.				
**Goal 8. Promote sustained, inclusive and sustainable economic growth, full and productive employment and decent work for all**						
8.5 By 2030, achieve full and productive employment and decent work for all women and men, including for young people and persons with disabilities, and equal pay for work of equal value	8.5.2 Unemployment rate, by sex, age and persons with disabilities	Divide the total number of unemployed (for a country or a specific group of workers) by the corresponding labour force (the sum of the total persons employed and unemployed) multiplied by 100.	In RSA in 2017:<br><br>Male – 26 %<br>Female – 29 %<br>Total – 27%	Please state your main weekly activity (employed).	Male – 42%<br>Female – 53%<br>Total – 49%<br><br>(These figures were derived by considering responses only from those who were 18 years and older).	
**Goal 11. Make cities and human settlements inclusive, safe, resilient and sustainable**						
11.1 By 2030, ensure access for all to adequate, safe and affordable housing and basic services and upgrade slums	11.1.1 Proportion of urban population living in slums, informal settlements, or inadequate housing	The number of urban individuals living in informal dwellings in South Africa divided by total	Percentage of urban population living in informal dwellings in 2017 in RSA: 12.2%	What type of dwelling is used?	3.0% of households approached were informal (i.e. 12 of 406 households).	

		number of the urban population multiplied by 100.				
**Goal 17. Strengthen the means of implementation and revitalize the Global Partnership for Sustainable Development**						
17.8 Fully operationalize the technology bank and science, technology and innovation capacity-building mechanism for least developed countries by 2017 and enhance the use of enabling technology, information and communications technology	17.8.1 Proportion of individuals using the Internet	Divide the total number of households using the internet by the total number of households multiplied by 100.	Percentage of households using the internet in RSA in 2017: 62%	Please state whether members of this household an of the following items (in working order): Assets – Internet	4% of households have access to internet	

Note: The numbering is the identical numbering used for the SDG goals, targets and indicators.

Definitions (exchange-rates.org)

1) **2015 International poverty line**: US$1.25/day
   - US $/ ZAR exchange rate June 2015: $1.00 = R 12.17
   - In June 2015 = $ 1.25/day = R 15.21/day
   - For a typical 30-day month, this translates to R456.38/month
2) **2018 International poverty line** = US$1.90/ day
   - US $/ ZAR exchange rate June 2015: $1.00 = R 13.71
   - In June 2015 = $ 1.25/day = R 17.14/day
   - For a typical 30-day month, this translates to R514.13/month
3) **National poverty line:** South Africa has developed three national poverty lines:

- a Food Poverty Line (FPL) at R441 per person per month;
- a Lower-bound Poverty Line (LBPL) at R647 per person per month and;
- an Upper-bound Poverty Line (UBPL) at R992 per person per month.

→ The country adopted the LBPL as the primary benchmark for monitoring poverty (SDG baseline report)

4) **Basic services:** Simultaneous access to water, sanitation, electricity and waste removal (http://www.seri-sa.org/index.php/what/basic-services)
