# Supplementary material for: Tracking Progress Towards the Sustainable Development Goals in Four Rural Villages in Limpopo, South Africa
Source: Ann Glob Health. 2021 Feb 15;87(1):16. doi: 10.5334/aogh.3139 (PMC7894382; doi:10.5334/aogh.3139)
Supplement: Table S2. — Comparing Giyani and South Africa SDG scores for targets for which indices could not be computed for official/direct comparison but were unofficially calculated for contextualization. [file agh-87-1-3139-s2.pdf]

Table S2. Comparing Giyani and South Africa SDG scores for targets for which indices could not be computed for official/ direct comparison but were unofficially calculated for contextualization.

SDG Target	SDG Indicator	Method of computation (RSA SDG reports)	South African score (based on RSA SDG reports)	Giyani Questionnaire item(s) providing relevant data in 2017	Giyani score
**Goal 1. End poverty in all its forms everywhere**					
1.2 By 2030, reduce at least by half the proportion of men, women and children of all ages living in poverty in all its dimensions according to national definitions	1.2.1A Number of social grants	This is a count of the number of social grants provided in a given year.	Number of social grants: 16 991 634	How many people in this household receive old age grant/ pension, childcare grant, disability grant or another grant?	Responses relevant to indicator: Overall number of grants reported to be received at the time of survey by all households: 823 (total sum of child-, old age-, disability- and other grants)<br><br>What does this mean: With 406 households surveyed, and a total of 823 grants received, there are numerous households which receive more than one type of grant.
1.3 Implement nationally appropriate social protection systems and measures for all, including floors, and by 2030 achieve substantial coverage of the poor and the	1.3.1D Percentage of population covered by social protection systems, distinguishing children, persons with disabilities or older persons.	The number of recipients of social assistance benefits and of the main contributory schemes divided by the total population, multiplied by 100.	Percentage of population covered by social protection system in 2016: 29%	How many people in this household receive old age grant/ pension, child support grant, disability grant or other type of grant?	This question cannot fully be answered, as we only know how many grants are received at a household level, not at an individual person level.<br><br>Old age grant: 43% of households had at least one member receiving this grant. Within each household, the percentage of people

vulnerable					receiving ranged from 0% – 100%.<br>Disability grant: 4% of households had at least one member receiving this grant. Within each household, the percentage of people receiving ranged from 0% – 50%.<br>Child grant: 70% of households had at least one member receiving this grant. Within each household, the percentage of people receiving ranged from 0% – 80%.<br>Other grant: 3% of households had at least one member receiving other grants.
1.4 By 2030, ensure that all men and women, the poor and the vulnerable, have equal rights to economic resources, as well as access to basic services, ownership and control over land and other forms of	1.4.1 Proportion of population living in households with access to basic services	Access to basic services, means access to water, sanitation, electricity and waste removal	Not included in SDG report	What type of toilet does the household mainly use?<br>How often does your waste get collected by local authority?<br>Where do you mainly get your drinking water?	The questionnaire does not enable us to identify whether the house is electrified or not (it can only be assumed by the indication of electrical appliances in a household). For this reason, "access to basic services" here focuses on access to water, sanitation and refuse removal.<br><br>36% of households surveyed had simultaneous access to

property, inheritance, natural resources, appropriate new technology and financial services, including microfinance					water, sanitation and refuse removal.
1.a Ensure significant mobilization of resources from a variety of sources, including through enhanced development cooperation, to provide adequate and predictable means for developing countries, least developed countries, to implement programmes and policies to end poverty in all its dimensions	1.a.3 Sum of total grants and non-debt-creating inflows directly allocated to poverty reduction programmes as a proportion of GDP	Sum of total grants (old age, disability and child support) as a proportion of total grants (old age, disability and child support) allocated to Limpopo province in 2017 in %	Not in SDG report<br>In Fact Sheet: Issue No.12 – December 2017<br>A Statistical Summary of Social Grants in South Africa: 14% of grants distributed in the country were distributed in Limpopo.	How many people in the household receive old age grant or pension, child support grant, disability grant or any other grant?	0.1 % of total national social grants allocated to Limpopo in 2017 were allocated to the households considered in the study.
**Goal 2. End hunger, achieve food security and improved nutrition and promote sustainable agriculture**					
2.2 By 2030, end	2.2.2 Prevalence of	The number of	Prevalence of	Body Mass Index	We know that 6% of

all forms of malnutrition, including achieving, by 2025, the internationally agreed targets on stunting and wasting in children under 5 years of age, and address the nutritional needs of adolescent girls, pregnant and lactating women and older persons	malnutrition (weight for height >+2 or <-2 standard deviation from the median of the WHO Child Growth Standards) among children under 5 years of age, by type (wasting and overweight)	children aged 0–4 years who are wasted (with weight-for-height below 2 SD from the reference median), divided by total number of children aged 0–4 years, multiplied by 100. A similar computation is done for overweight (weight-for-height above 2 SD from the reference median).	Malnutrition Among Children (U5Yrs): 2016: <br>Malnutrition: 16% Wasting: 3% Overweight: 13% <br>*Comment: Malnutrition consists of both wasting and overweight	calculated using measured weight and height.	respondents are defined "underweight", given their BMI. We do not know what the BMI is for children under the age of 5 in Giyani.
**Goal 3. Ensure healthy lives and promote well-being for all at all ages**					
3.a Strengthen the implementation of the World Health Organization Framework Convention on Tobacco Control in all countries, as appropriate	3.a.1 Age-standardized prevalence of current tobacco use among persons aged 15 years and older	The number of tobacco smokers aged 15 years and above divided by the population aged 15 years and above multiplied by 100.	Prevalence of tobacco use by sex (15 years and older) in 2016: 37 % male smokers 7 % female smokers	Does anyone in the household use tobacco?	6% of those surveyed smoked tobacco. Of those who smoked, 23% were women and 77% were men. We did not ask about the ages of the people in the households who smoked, and so we cannot directly compare this indicator.
**Goal 4. Ensure inclusive and equitable quality education and promote lifelong learning opportunities for all**					
4.1 By 2030, ensure that all girls and boys complete	4.1.1 Proportion of children and young people (a) in grades	NA	NA	What is the highest achieved education qualification?	13% of individuals were not educated at all. 29 % of individuals reportedly

free, equitable and quality primary and secondary education leading to relevant and effective learning outcomes	2/3; (b) at the end of primary; and (c) at the end of lower secondary achieving at least a minimum proficiency level in 9i) reading and (ii) mathematics, by sex.				completed primary education. 41% of individuals in the households considered completed secondary education.
**Goal 5. Achieve gender equality and empower all women and girls**					
5.b Enhance the use of enabling technology, information and communications technology, to promote the empowerment of women	5.b.1 Proportion of individuals who own a mobile telephone, by sex	The number of individuals who own a mobile telephone in working order divided by total population multiplied by 100.	Percentage of individuals who own a mobile telephone, by sex: Male - 65% Female – 67%	Please state whether members of this household an of the following items (in working order): Asset – Mobile Phone.	79% of households own a cell phone, but we don't know whom they belong to in the household, and so we cannot allocate the cell-phones according to sex.
**Goal 7. Ensure access to affordable, reliable, sustainable and modern energy for all**					
7.1 By 2030, ensure universal access to affordable, reliable and modern energy services	7.1.1 Proportion of population with access to electricity	7.1.1 Number of individuals with access to electricity divided by total number of individuals in South Africa multiplied by 100.	In 2019: 95% of the population had access to electricity	During very cold weather, do you use any of the following systems to heat your home? What fuel do you usually use for cooking?	We do not know how many households are electrified here (as we did not ask the question "does your households have access to electricity?"). We know that some households which have electrical appliances probably have access to electricity. These households also burn wood for heating and/or cooking purposes, and so it can be assumed that some

					form of energy stacking/ fuel stacking takes place. However, we cannot assume that those households which do not own electrical appliances or household which use dirty fuel for cooking or heating do not have electricity. 22% of households reportedly used electricity for cooking and 13% used and electric heater.
7.1 By 2030, ensure universal access to affordable, reliable and modern energy services	7.1.2 Proportion of population with primary reliance on clean fuels and technology	7.1.2D: Percentage of the population that uses solar energy as their main source of energy	In 2017 less than 1% of the population used solar energy for cooking, lighting, heating of water or space heating.	During very cold weather, do you use any of the following systems to heat your home? What fuel do you usually use for cooking?	As this question is similar to the above, it is an index we are unable to quantify in this survey. We know that 78% of households use wood for cooking (the remaining 22% use electricity). This means that the greater proportion of the households, though they might have access to electricity (the "cleaner fuel"), they do not necessarily make use of it (this could be for reasons related to affordability, for example).
**Goal 8. Promote sustained, inclusive and sustainable economic growth, full and productive employment and decent work for all**					
8.6 By 2020, substantially reduce the proportion of youth not in	8.6.1 Proportion of youth (aged 15–24 years) not in education, employment or	"Not in employment and not in education or training" (NEET) rate is calculated	NEET for 2016 = 31%	Please state your main weekly activity (employment etc.).	We are only able to calculate what proportion of those aged 15-24 is unemployed (24%).

employment, education or training	training	as the number of youth aged 15–24 years who are not in employment, not in education or training, divided by the population aged 15–24 years multiplied by 100.			
**Goal 10. Reduce inequality within and among countries**					
10.2 By 2030, empower and promote the social, economic and political inclusion of all, irrespective of age, sex, disability, race, ethnicity, origin, religion or economic or other status	10.2.1 Proportion of people living below 50 per cent of median income, by sex, age and persons with disabilities	Proportion of people living below 50 percent median income by age group.	2015:<br><br>0-17yrs: 44%<br>18-24yrs: 38%<br>25-34yrs: 29%<br>35-44yrs: 24%<br>45-54yrs: 23%<br>55-64yrs: 23%<br>65+yrs: 21%	What is the average monthly income for this household- excluding grant and pensions	We are unable to calculate this as we have categorical income data.
**Goal 11. Make cities and human settlements inclusive, safe, resilient and sustainable**					
11.6 By 2030, reduce the adverse per capita environmental impact of cities, including by	11.6.1 Proportion of urban solid waste regularly collected and with adequate final discharge out of total urban solid	11.6.1D: Percentage of municipal waste generated and recycled	In 2017 8% of municipal waste generated and recycled	Where is your refuse container (bin) located?<br>Does your bin have a properly fitted lid?	Our survey does not enable us to answer this question (adequate final discharged out of total urban solid waste generated) – but we know that 63% of households do not

paying special attention to air quality and municipal and other waste management	waste generated, by cities			How often does your waste get collected by local authority?<br>If your refuse is not collected, how do you dispose it	have formal refuse collection

Note: The numbering is the identical numbering used for the SDG goals, targets and indicators.
